# Circulating microRNA profiles of Ebola virus infection

**DOI:** 10.1038/srep24496

**Published:** 2016-04-21

**Authors:** Janice Duy, Jeffrey W. Koehler, Anna N. Honko, Randal J. Schoepp, Nadia Wauquier, Jean-Paul Gonzalez, M. Louise Pitt, Eric M. Mucker, Joshua C. Johnson, Aileen O’Hearn, James Bangura, Moinya Coomber, Timothy D. Minogue

**Affiliations:** 1 Diagnostic Systems Division, U.S. Army Medical Research Institute of Infectious Diseases, Fort Detrick, Frederick, MD, USA; 2 Virology Division, U.S. Army Medical Institute of Infectious Diseases, Fort Detrick, Frederick, MD, USA; 3 Metabiota, Kenema, Sierra Leone; 4 Metabiota, Washington, DC, USA

## Abstract

Early detection of Ebola virus (EBOV) infection is essential to halting transmission and adjudicating appropriate treatment. However, current methods rely on viral identification, and this approach can misdiagnose presymptomatic and asymptomatic individuals. In contrast, disease-driven alterations in the host transcriptome can be exploited for pathogen-specific diagnostic biomarkers. Here, we present for the first time EBOV-induced changes in circulating miRNA populations of nonhuman primates (NHPs) and humans. We retrospectively profiled longitudinally-collected plasma samples from rhesus macaques challenged via intramuscular and aerosol routes and found 36 miRNAs differentially present in both groups. Comparison of miRNA abundances to viral loads uncovered 15 highly correlated miRNAs common to EBOV-infected NHPs and humans. As proof of principle, we developed an eight-miRNA classifier that correctly categorized infection status in 64/74 (86%) human and NHP samples. The classifier identified acute infections in 27/29 (93.1%) samples and in 6/12 (50%) presymptomatic NHPs. These findings showed applicability of NHP-derived miRNAs to a human cohort, and with additional research the resulting classifiers could impact the current capability to diagnose presymptomatic and asymptomatic EBOV infections.

Rapid and accurate diagnosis of highly transmissible, lethal illnesses such as Ebola virus disease (EVD) is critical to restricting pathogen spread and to applying appropriate therapeutic strategies. As demonstrated by the recent EVD outbreak in Western Africa, early detection and confirmation of suspected cases are essential to halting disease spread^1^. Identification of EVD is especially important as initial symptoms such as fever, weakness, dizziness, sore throat, and headache^2^ mimic those of endemic diseases such as dengue fever, Lassa fever, and malaria^3^,^4^. Furthermore, survivors of the disease may remain infectious as several studies found virus in semen^5^,^6^, vaginal secretions^7^, breast milk^8^, and the aqueous humor of the eye^9^ for weeks to months post-recovery, after the pathogen was no longer detectable in blood. Additionally, one patient with high viral titer and actively shedding virus prior to the appearance of clinical symptoms was recently reported^10^.

Current diagnostics rely on identifying Ebola virus (EBOV) in blood samples by targeting viral antigens using enzyme immunoassays or by amplifying specific viral sequences through quantitative reverse transcriptase polymerase chain reaction (RT-PCR)^11^, with PCR results available 1–2 days earlier than corollary immunoassays. However, these tests may be unreliable until 3–4 days after symptom onset^12^,^13^. Because time between infection and symptom onset ranges from 1–15 days^14^, it is plausible that asymptomatic or mildly symptomatic hosts can unknowingly transmit disease through bodily fluids or tissues, and possibly through aerosolized particles^15^,^16^. As well, convalescent patients who have cleared virus from circulation may still be harboring infectious EBOV in immunologically protected sites such as the brain and central nervous system, the eye, and the male genital tract^17^.

Presymptomatic diagnostic efforts currently focus on host responses to pathogens as molecular induction of host machinery occurs before clinical presentation and detection of pathogen. Specifically, analyzing changes in host gene expression levels could potentially generate pathogen-specific profiles as in theory, different infectious agents will elicit distinct immune reactions. For instance, longitudinal studies of gene expression retrospectively differentiated symptomatic and asymptomatic influenza A virus infections based on host expression profiles^18^. Subsequent use of a downselected host gene expression profile classifier distinguished between two influenza A subtypes and correctly predicted infections (92.3% accuracy) in symptomatic patients during the 2009 flu season^19^. In this context, EBOV infections provoke massive host immune responses that can be leveraged for earlier diagnosis^20^. To date, molecular host responses to EVD are not utilized for disease detection, and such a test would significantly impact EVD treatment and containment.

Genomic DNA and messenger RNA (mRNA) are common targets for pathogen signature discovery; however, microRNAs (miRNAs) are emerging as ideal diagnostic targets. miRNAs are short (~22 nt) non-coding RNA sequences that control gene expression through binding specific mRNAs. This interaction results in inhibition of translation or initiation of RNA degradation^21^. In comparison to mRNAs or proteins, fewer discrete miRNAs have been identified as each miRNA can pleiotropically regulate several gene expression networks^22^. Thus, miRNA expression patterns are computationally less complex to analyze than global gene expression. miRNAs are readily accessible for interrogation due to release into circulation through active secretion or cell death (apoptosis, necrosis)^23^. In this context and in terms of diagnostic relevance, miRNAs are remarkably robust and survive nuclease degradation, with previous studies documenting detection in various bodily fluids such as blood (serum, plasma), cerebrospinal fluid, breast milk, urine, and saliva^24^. Several groups exploited these features of miRNAs to uncover disease signatures from diverse cancer types^25^ and infections of bacterial^26^ and viral^27^ origin. Most recently, Sheng *et al.*^28^ showed 18 miRNAs differentially expressed in human umbilical vein endothelial cells (HUVECs) infected with EBOV glycoprotein (GP)-expressing adenovirus. In addition, inhibitors of three of these sequences could prevent GP-induced cytotoxicity *in vitro*, underscoring the potential therapeutic benefits from this line of inquiry.

In this study, we analyzed the expression of 752 circulating miRNA sequences in archived plasma from rhesus macaques exposed to EBOV infected either through intramuscular injection or aerosol inhalation. We identified miRNAs that showed significant changes in abundance during lethal EBOV infection for each group. We also profiled 15 cell-free blood samples from human EBOV cases during the 2014 outbreak in Western Africa. We found 15 miRNAs correlated with viral titer in both rhesus macaque as well as human samples. As a proof of concept for a host miRNA-driven diagnostic, we identified an eight-miRNA classifier predictive of acute infection with high accuracy in both NHPs and humans, and this classifier also identified half of the presymptomatic macaque hosts.

## Results

### Ebola virus infection in rhesus macaques

**Figure 1:**
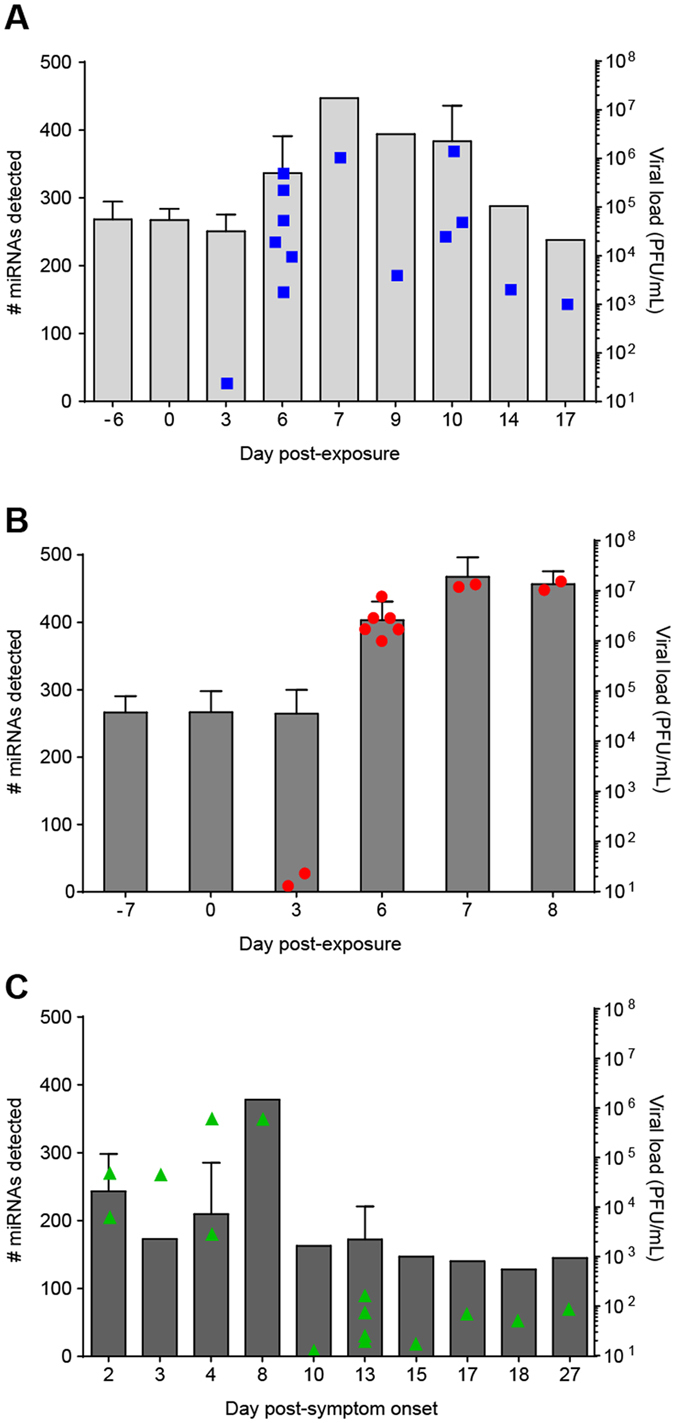
miRNA profiles and viral loads during Ebola virus infection. Numbers of circulating miRNAs detected during EBOV infection (bars) overlaid with viral loads (filled symbols) for **(A)** intramuscularly (IM)-challenged rhesus macaques, **(B)** aerosol-challenged rhesus macaques, and **(C)** human samples. miRNA values are given as the mean ± SD, while viral loads are plotted individually. Viral loads were calculated from serial dilutions of the EBOV/Kikwit challenge stock, measured in PFU/mL based on plaque assay.

NHP samples used in this work were acquired from an unrelated study. For intramuscular (IM) challenge, animals (n = 6) received an average dose of 245 PFU of EBOV/Kikwit (Ebola virus/H.sapiens-tc/COD/1995/Kikwit-9510621), as determined by back titration by plaque assay. Circulating virus was detectable in one animal at day 3 and in all NHPs starting on day 6; viral titers ranged from 10^3^–10^6^ PFU/mL through death/euthanasia (Fig. 1A). Using hourly analysis, the median survival time was 9.36 days; one animal was euthanized on day 7, two animals succumbed on day 9, two on day 10, and one NHP was euthanized on day 17 post-challenge (Supplementary Fig. S1). For aerosol challenge, animals (n = 6) received an average dose of 1367 PFU of EBOV/Kikwit, as determined by back titration by plaque assay. Circulating virus (~10^6^ PFU) was detected in all animals by day 6 and remained high until time of death/euthanasia (Fig. 1B). The median survival time was 7.23 days; two animals succumbed to the disease on day 7, and the remaining NHPs were euthanized on day 7 and day 8 once euthanasia criteria were met (two animals each day, Supplementary Fig. S1). EBOV was detectable in the plasma of two NHPs starting at day 3 (~20 PFU/mL, Fig. 1B). Clinical presentation of disease and serum chemistry levels (Supplementary Fig. S2) were consistent with previously reported findings for EVD in IM-challenged^29^,^30^ and aerosol-exposed^31^,^32^ rhesus macaques.

### miRNA profiles over the course of EVD progression in nonhuman primates

We assayed longitudinal plasma samples using a PCR-based detection system to profile the cell-free blood miRNA response to EBOV infection. This analysis found that numbers of circulating miRNAs increased dramatically during infection (Fig. 1A,B, bars). Among IM-challenged NHPs, an average of 262 miRNAs was detected in uninfected and presymptomatic animals (days −6, 0, and 3). This number rose to over 336 miRNAs by day 6, with increasing numbers of miRNAs present in plasma as the disease progressed (Fig. 1A). To investigate whether this increase in the number of circulating miRNAs was related to the amount of EBOV present, we performed a Pearson’s correlation on the two variables. This analysis showed that miRNA counts correlated highly with viremia (r = 0.82, p-value = 1.76E-8). A total of 478 miRNAs (of the 752 included in the Exiqon Human panels I+II) were detected over the course of infection (data not shown). Of these, two-step regression analysis using maSigPro^33^ showed 41 (8.6%) miRNAs differentially present over baseline. Thirty-two miRNAs increased in abundance through day 6, while 9 decreased over the same time period (Supplementary Table S2).

Similarly, among aerosol-exposed NHPs, PCR assays detected a mean of 265 plasma miRNAs prior to symptom onset (day −7, day 0, and day 3). This number rose to over 403 miRNAs among infected, symptomatic animals (day 6), and time of death or euthanasia yielded additional miRNAs (day 7 and day 8). Again, the number of miRNAs in plasma correlated highly with viral load (Pearson’s r = 0.95, p-value = 2.99E-14). A total of 516 miRNAs were detected over the aerosol disease timecourse. Two-step regression determined a large percentage of these miRNAs, 40.1% (207 sequences) as differentially present (≥2 absolute fold change over the baseline values). Of this number, 138 increased in abundance through day 6, and 69 miRNAs decreased relative to the baseline (Supplementary Table S3).

**Figure 2:**
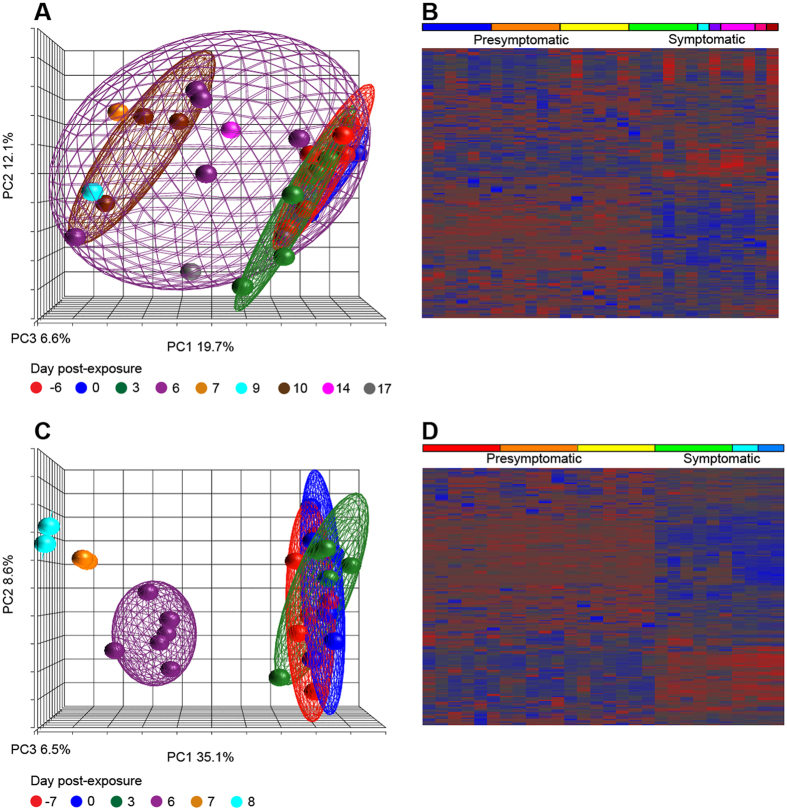
Global miRNA-derived characteristics of Ebola virus-infected NHPs. Principal components analysis (PCA) and expression heatmaps constructed using miRNA profiles during the course of EBOV infection. For intramuscularly-challenged rhesus macaques, the PCA graph and heatmap are shown in **(A)** and **(B)**, respectively. Correspondingly, the PCA graph and heatmap are shown for aerosol-challenged animals in **(C, D)**. Ellipsoids were drawn to highlight NHP clusters for each blood draw day in PCA graphs. Hierarchical clustering was used to group miRNAs in heatmaps. Raw miRNA values were normalized to the average Cq per plate and log_2_ transformed prior to clustering.

Disease progression and host miRNA response differed between the two NHP groups based on viral loads at each blood draw (Fig. 1A,B, filled symbols), principal components analysis (PCA), and clustered miRNA heatmaps (Fig. 2). For the IM cohort, the average correlation of miRNA abundances for individual macaques was high (Pearson’s r > 0.87) prior to symptom onset; however, at day 6 this number dropped to 0.75 and then increased with clinical disease progression (data not shown). PCA results showed that uninfected to presymptomatic as well as late-course disease samples formed distinct groups (Fig. 2A). However, day 6 (symptomatic) samples spanned both presymptomatic and terminal disease PCA spaces. These trends are also reflected in the miRNA heatmap (Fig. 2B). In contrast, viral loads in the aerosol-infected NHPs were much higher by day 6 and varied little through time of death. Average pairwise correlation coefficients of these macaques were also uniformly high (Pearson’s r > 0.96) over the course of infection, demonstrating the consistency of miRNA abundances in the animals of this group (data not shown). In this cohort, clear distinctions can be seen among presymptomatic, symptomatic, and time of death/euthanasia samples visualized with PCA (Fig. 2C) and a clustered heatmap (Fig. 2D).

Comparison of significant miRNAs from two-step regression analysis showed that 36 sequences were common to both aerosol- and IM-challenged NHPs, with 28 and 8 miRNAs increasing and decreasing, respectively, over time. One sequence, hsa-miR-708-3p, increased in IM challenge but declined in the aerosol cohort (Supplementary Tables S2 and S3).

### Circulating miRNA profiles of human samples from 2014 West African Ebola virus outbreak

We next wanted to determine if NHP-identified miRNAs translated to EBOV-infected human patients. Fifteen cell-free human blood samples collected from Sierra Leone in 2014 were profiled for miRNAs and viral load using real-time RT-PCR. All samples were confirmed EBOV-positive using an in-house RT-PCR assay^11^. This analysis showed elevated miRNA counts (mean of 215 sequences) for cell-free blood drawn up to 4 days after patient-reported onset of symptoms, when corresponding viral loads were >1000 PFU/mL (acute infection). A total of 346 sequences were identified in this timeframe. Starting at day 10 post symptom onset, when viremia was measured at <200 PFU/mL (Fig. 1C), the average number of miRNAs detected decreased to 156, with 236 total miRNAs identified. Of these, 110 were unique to acute infection. We observed highest circulating miRNA abundances in the one sample collected on day of death (378 sequences, day 8). Of these, 208 miRNAs were shared with all samples, and 71 miRNAs were also present only in acutely infected samples. A further 89 miRNAs were unique to this individual at time of death. As was observed in the NHP groups, numbers of detectable miRNAs in these patients correlated with viremia (Pearson’s r = 0.78, p-value = 5.69E-4).

Longitudinal samples from the one patient with two separate blood draws yielded the same number of miRNAs (140) detected 4 days apart, 13 and 17 days after reported onset of illness. Viral load was 19.6 and 69.3 PFU/mL on these days, respectively. Ninety-five sequences (67.9%) were common to this patient on both days, with 45 sequences unique to each timepoint.

### miRNAs significantly correlated with viral load in NHPs and human subjects

**Figure 3:**
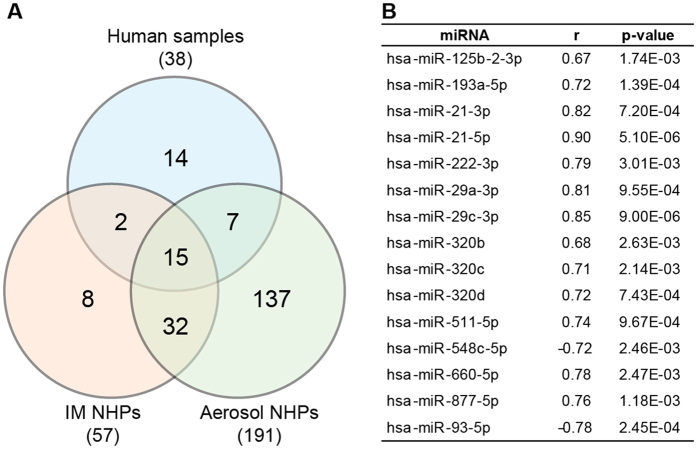
miRNAs correlated with circulating Ebola viral load. **(A)** Numbers of miRNAs correlated with EBOV measured using real-time RT-PCR for NHPs and humans with Pearson’s |r| > 0.6, p-value < 0.01. **(B)** List of 15 viral load-associated miRNAs shared between both NHP groups and human samples. The Pearson’s r value given is the average of the three groups.

Similar to initial experiments, we further explored whether individual miRNA abundances also correlated with viral titer. We calculated Pearson’s correlations for each dataset, with cutoffs set to |r| > 0.6, p-value < 0.01. IM-challenged NHPs, aerosol-challenged NHPs, and human cohorts yielded 57, 191, and 38 sequences meeting these criteria, respectively. Of these, 47 miRNAs were common to both macaque groups, with 15 sequences shared between the two species (Fig. 3). All miRNAs except hsa-miR-548c-5p and hsa-miR-93-5p increased with circulating viral titer. Comparison of these viral load-correlated sequences with the 36 miRNAs significantly differentially present in both NHP groups (determined by two-step regression analysis) identified 12 miRNAs in both sets: hsa-miR-193a-5p, -21-3p, -21-5p, -222-3p, -29a-3p, -29c-3p, -320b, -320c, -320d, -548c-5p, -660-5p, and -877-5p.

### Potential Ebola virus infection diagnostic miRNA model

**Figure 4:**
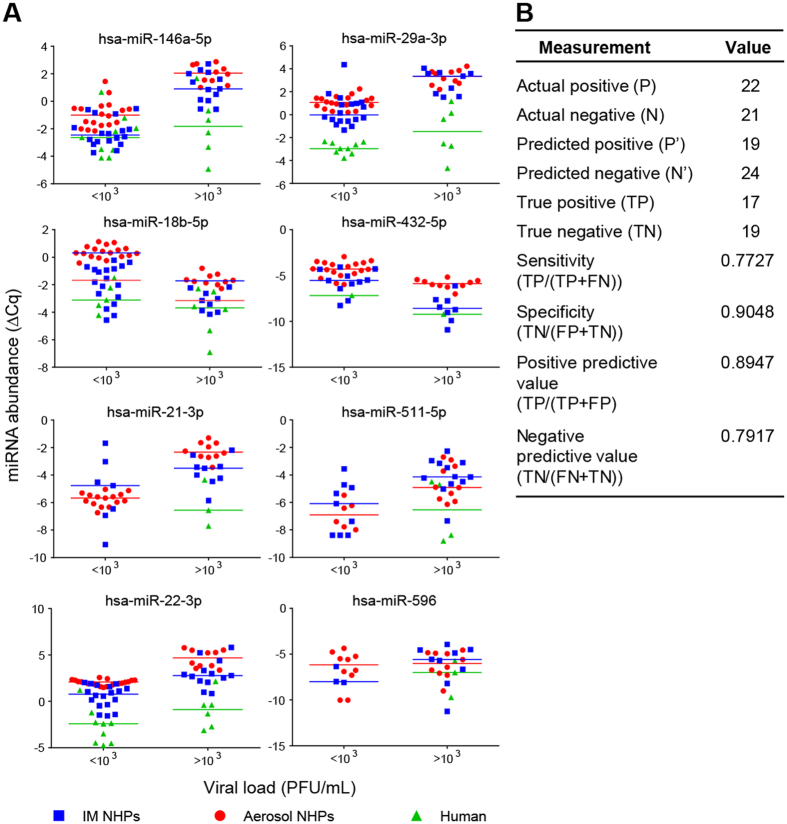
Select circulating miRNAs can identify acute Ebola virus infection in NHPs and humans. Eight host-derived miRNAs were selected by a support vector machine (SVM) classifier trained on intramuscularly-challenged rhesus macaques (n = 6) to identify EBOV infection. **(A)** Relative miRNA abundances (ΔCq) are shown for each classifier miRNA, separated by viral load. Individual points are plotted for each sample where the given miRNA was amplifiable, with lines are drawn to show the median value for each cohort. **(B)** Classifier performance metrics calculated from testing aerosol NHP and human samples combined (n = 43). Performance parameters are described briefly in the main text.

To further support the diagnostic relevance of circulating miRNAs for EVD, we used samples from the NHP and human cohorts separately to construct a classifier for EBOV infection. We tested a number of classifier algorithms, and the best-performing method (Supplementary Table S4) was a support vector machine (SVM) trained on the IM-challenged NHP data, composed of eight miRNAs. SVM is a method where data classes (in this instance, infected or uninfected) are passed on to the algorithm for training (supervised learning). Data points are transformed to a higher-order (nonlinear) feature space where the data classes can be clearly separated. Class selection is achieved by maximizing the distance between the transformed data groups with the classification function trained on data points (called support vectors) closest to the separation boundary^34^,^35^. Previous studies show this type of classifier to be highly accurate (out of 179 tested) for a wide variety of datasets^36^, and it surpassed other methods for gene expression analysis^37^,^38^. In addition, SVMs can extract relevant classification information even when the number of available samples is low relative to targets assayed, as in microarray profiling experiments with thousands of gene probes^39^,^40^. In this work, the SVM method selected hsa-miR-146a-5p, hsa-miR-18b-5p, hsa-miR-21-3p, hsa-miR-22-3p, hsa-miR-29a-3p, hsa-miR-432-5p, hsa-miR-511-5p, and hsa-miR-596 as an EVD classifier. Six of the miRNAs increased in abundance with higher viral load (>1000 PFU), while miR-18b-5p and miR-432-5p declined (Fig. 4A). These trends were consistent among the macaque and human cohorts with the exception of miR-21-3p, -511-5p, and -596, which were not detected in human samples with low viral titers.

With the IM NHP training set, the classifier yielded correct categorization of 28/31 (90%) of samples. Specifically, this classifier demonstrated correct identification of 50% (3/6) of presymptomatic infected NHPs as well as 100% of acutely infected, symptomatic samples. Since the classifier was developed using the IM NHP cohort as the training set, we further tested model performance using the aerosol-challenged NHP and human samples as testing sets. For the aerosol NHP samples, the miRNA classifier correctly distinguished 24/28 (86%) samples, with 50% (3/6) of presymptomatic and 100% of symptomatic animals identified. Similarly, using the human cohort, the NHP-trained model correctly predicted the infection status of 12/15 samples (80%), including 67% (4/6) of acutely infected human patients.

Blinded testing of aerosol NHP and human samples combined and randomized (n = 43) further supported the miRNA classifier performance (Fig. 4B). Downselection of miRNAs retaining only sequences present in at least one group yielded a total of 360 miRNAs. This analysis showed accurate classification of 36/43 samples (accuracy of 84%). The sensitivity of the classifier, or proportion of true positives that were correctly identified, was 77%. The specificity of the classifier, or proportion of correctly identified true negatives, was 90%. The positive predictive value of the classifier, which is the probability that a sample identified as positive is a true positive, is 89%. Similarly, the negative predictive value, or probability that a sample identified as negative is a true negative, is 79%. Receiver operating characteristic (ROC) analysis yielded an area under curve (AUC) of 0.8387; this value is a measure of the accuracy of classifying a sample correctly, with perfect classification yielding AUC = 1. The model performed better with acute infections, which were correctly identified in 14/16 samples (88%). Only one uninfected sample was misclassified out of 12 (83% correct). In addition, miRNA profiling also showed correct determination of 50% (3/6) presymptomatic samples (day 3 NHPs from the aerosol cohort).

### Predicted gene targets and pathway analyses of significant miRNAs

**Figure 5:**
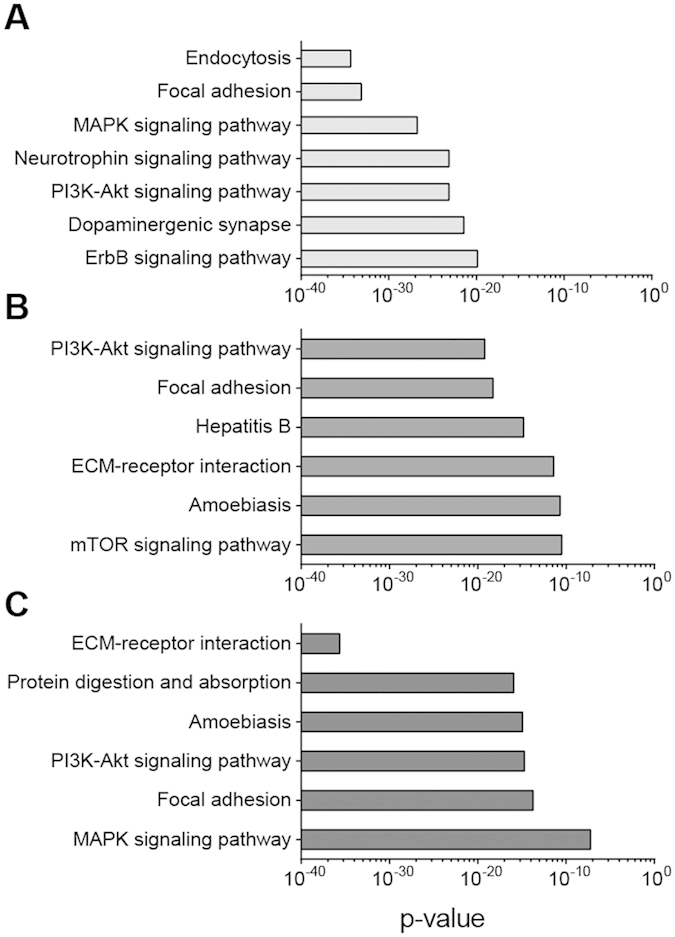
KEGG pathways implicated by significant miRNAs in Ebola virus infection. Top overrepresented non-cancer Kyoto Encyclopedia of Genes and Genomes (KEGG) pathways from DIANA-mirPath v3.0 analysis, based on miRNAs **(A**) significantly present in both IM- and aerosol-challenged rhesus macaques, **(B**) correlated with viral load in rhesus macaques and humans, and **(C**) selected by best-performing support vector machine model. Significant pathways were determined using the union of predicted miRNA target genes.

We next explored how miRNA species significantly present during EVD might putatively fit into the framework of what is currently known about EBOV pathogenesis. In this context, DNA Intelligent Analysis (DIANA)-miRPath v3.0^41^ analysis showed predicted miRNA gene targets. Specifically, we examined the 36 miRNAs common to both IM and aerosol EBOV-infected NHP groups, the 15 transcripts correlated to circulating virus in humans and NHPs, and the 8 miRNAs used as a classifier. DIANA-miRPath v3.0 analysis using these predicted mRNAs calculated significantly overrepresented biological pathways. The top non-cancer-associated enriched Kyoto Encyclopedia of Genes and Genomes (KEGG) pathways for each miRNA group are shown in Fig. 5. Focal adhesion and the phosphoinositide 3-kinase (PI3K)-Akt signaling pathways are common to all three sets of predicted miRNA targets, while extracellular matrix (ECM)-receptor interaction and amoebiasis are shared by miRNAs associated with viral load and predictors of acute EBOV infection.

## Discussion

State-of-the art diagnostic methods for EBOV infection rely on direct detection of virus signatures through PCR or immunoassay. These tests determine discharge for convalescent patients in the current outbreak; specifically, individuals must be asymptomatic and blood virus levels should be undetectable for 2–3 sequential PCR assays (Cq ≥ 40)^42^. However, documented cases of symptomless EBOV infection showed no detectable levels of circulating virus in over a third of the cases^43^. In contrast, this same study showed that all asymptomatic cases presented strong inflammatory responses to the pathogen, demonstrating the value of assessing host-based markers of acute disease or previous infection. To that end, we profiled miRNAs in EBOV-infected rhesus macaques and humans for diagnostic applications due to miRNA accessibility, robustness, and biological informativeness.

We demonstrated here for the first time that EBOV infection is accompanied by changes in circulating host miRNA populations. In this context, the numbers of plasma miRNAs increased markedly with disease progression, and a sizeable proportion of these sequences were differentially present over time. In addition, these data led to a preliminary NHP-based model of eight host-encoded miRNAs that identified acute EBOV infection with high accuracy, as well as half of the presymptomatic animals. Evaluation of this model with human samples from the 2014 EBOV outbreak in Sierra Leone confirmed the translation of NHP studies to human cases.

Significant changes in circulating miRNAs were observed for both IM- and aerosol-challenged rhesus macaques, with distinct miRNA species and abundances correlated with viral load. Interestingly, similar trends were uncovered in cell-free blood samples from human EVD cases, with correlations between viremia and numbers of discrete species as well as their abundances. While the lack of matched controls and longitudinal collection timepoints precludes determination of differentially expressed miRNAs during EVD in humans, we identified some potential targets for further study. In particular, our data showed 15 miRNAs associated with viral titer in rhesus macaques and humans infected with separate variants of *Zaire ebolavirus.* RNAhybrid analysis^44^ predicted binding of these sequences to bind to both EBOV/Kikwit (GenBank: JQ352763.1) and EBOV/Makona (GenBank: KM034557.1). Indeed, previous studies and a growing body of evidence show that cellular miRNAs profoundly influence viral replication and pathogenesis, so it is intuitive that miRNAs could impact EVD progression.

Diverse cellular miRNA species affect viral replication by restricting expression as in the case of primate foamy virus type 1^45^, HIV-1^46^,^47^, and vesicular stomatitis virus^48^, or by enhancing production, as with hepatitis C virus^49^. To date, hsa-miR-122-5p is the only miRNA reported as biomarker of EBOV infection in rhesus macaques^50^. This miRNA was induced in higher levels in non-survivors compared to anticoagulant-treated survivors, and was part of a minimal set of 20 molecular probes that determined EVD outcome in the NHP model. Consistent with these results, we observed elevated levels of miR-122-5p in all acutely infected NHPs and some human samples, but could not correlate its abundance with clinical outcome in our datasets. In another recent study, human umbilical vein endothelial cells (HUVECs) were infected with EBOV glycoprotein-expressing adenovirus, and the authors identified 18 miRNAs differentially expressed between 3 and 24 hours post-infection using RNA-seq^28^. From these, they discovered that inhibition of miR-1246, miR-196b-5p, and miR-320a, which were upregulated in infected cells, could prevent cell death. In our investigations, miR-1246 was not part of the PCR array used, while miR-196-5p was present in only one human sample (drawn closest to time of death) and not detected in any NHP samples. However, miR-320a, along with other members of the miR-320 family (miR-320b, -320c, and -320d) increased in abundance during infection. miR-320 is an anti-angiogenic factor that inhibits endothelial cell growth and migration^51^.

We performed gene target predictions and pathway overrepresentation analysis to explore biological functions potentially regulated by miRNAs significantly present during EBOV infection. Perhaps unsurprisingly, ECM-receptor interaction, focal adhesion, and the PI3K-Akt signaling pathways were significantly enriched. Endocytosis, which is the known mechanism of entry of the filovirus^52^, is putatively regulated by miRNAs significantly present during EBOV infection in NHPs. The PI3K-Akt pathway is activated early in this process and is critical to viral entry since inhibition of the cascade arrested further pathogen trafficking^53^. Focal adhesion and ECM-receptor interaction are pathways substantially affected by EVD, since the virus induces dysregulation of endothelial cells^54^,^55^,^56^. These findings putatively implicate miRNA roles and participation in EBOV entry, pathogenesis, and disease development. If confirmed in subsequent studies, these miRNAs may also represent potential therapeutic targets.

Research described in this work establishes the relevance and utility of circulating miRNAs for identifying EBOV infection in presymptomatic and asymptomatic individuals. While the diagnostic gold standard is the PCR detection of virus in blood, several studies have demonstrated that infectious EBOV may resurface from immunological sanctuary sites months after patients are discharged^5^,^6^,^7^,^8^,^17^,^57^. A host-based indicator of disease, such as the one presented in this work, may be most useful for post-exposure monitoring of EBOV patient household members and contacts, establishing effective traveler screening guidelines^58^, and for deploying military personnel^59^. Asymptomatic individuals, who could be recruited as caregivers in healthcare-scarce settings^60^, may also be identified using this method.

The eight-miRNA classifier presented here accurately identified acutely infected individuals, and half of presymptomatic NHP hosts. The classifier was developed from macaques injected with EBOV, which better simulates transmission through skin break or accidental needle stick^61^. Additionally, the animals in this cohort displayed diversity in molecular attributes, which is typical of an infected population. While these eight miRNAs have known functions in innate immune responses, further work is required to elucidate their roles in EBOV pathogenesis. Similarly, to determine if this classifier accurately represents an EVD-specific signature, additional studies are required. These would consist of larger NHP and human sample sets representing infections not only from EBOV but also from other viral and bacterial pathogens with similar clinical symptoms to define the specificity of the EBOV infection classifier and to characterize new miRNA-based identifiers for other diagnostic contexts. As well, our investigation here was constrained to miRNAs released into plasma or serum, but previous studies noted EBOV-induced gene expression changes as early as 1–2 days post-infection in PBMCs^62^. Intuitively, this observation suggests a likely measurable shift in miRNAs in these cell types. These caveats aside, this work shows that miRNAs are potential diagnostic candidates via a proof of concept acute EVD classifier while also establishing the potential basis for presymptomatic or asymptomatic diagnosis of the disease.

## Materials and Methods

### Ethics statement and disclosures

Rhesus macaque plasma samples were archived samples not collected specifically for this study. NHP experiments and procedures were approved by the U.S. Army Medical Research Institute of Infectious Diseases (USAMRIID) Institutional Animal Care and Use Committee (IACUC) and were carried out in compliance with the regulations outlined in the USDA Animal Welfare Act (PHS Policy) and other federal statutes and regulations relating to animals and experiments involving animals. The facility where this research was conducted is accredited by the Association for Assessment and Accreditation of Laboratory Animal Care, International and all animal work done adhere to the conditions specified in the Guide for the Care and Use of Laboratory Animals^63^. Animals were given enrichment (including toys and mirrors) regularly as recommended by the Guide for the Care and Use of Laboratory Animals. Food was provided (commercial biscuits, fruit), and animals were checked at least daily according to the protocol. All efforts were made to minimize painful procedures; the attending veterinarian was consulted regarding painful procedures, and animals were anesthetized prior to phlebotomy. Following the development of clinical signs, animals were checked multiple times daily. When clinical observations and scores of animals reached defined levels based on the approved IACUC protocol (scores based on a combination of responsiveness, recumbency, and clinical signs), animals were euthanized by exsanguination following deep anesthesia and administration of a pentobarbital-based euthanasia solution to minimize pain and distress. All animals were housed at USAMRIID.

Research on human subjects was conducted in compliance with United States Department of Defense, federal, and state statutes and regulations relating to the protection of human subjects, and adheres to principles identified in the Belmont Report^64^. All data and human subjects research were gathered and conducted for this publication under an Institutional Review Board approved protocol.

De-identified human serum and plasma samples from individuals infected with EBOV in the Republic of Sierra Leone, West Africa were used in this study. These samples were determined by the institutional Office of Human Use and Ethics to be Not Human Subject Research. All samples were collected and de-identified in Sierra Leone at Kenema Government Hospital. The samples had indirect identifiers when we received them. None of the samples were collected for this study; this study was an additional use of the samples collected for another purpose. Samples were collected during the outbreak to perform emergency diagnostics. In this context, the need for written informed consent was waived by the Ministry of Health and Sanitation of the Republic of Sierra Leone.

The opinions, interpretations, conclusions, and recommendations contained herein are those of the authors and are not necessarily endorsed by the United States Army.

### NHP exposure to Ebola virus

#### NHP Ebola models via intramuscular and aerosol routes

Samples used in this work were archived samples not collected for the purpose of this study, but were from the *in vivo* characterization of a challenge material working stock as described here in brief. The USAMRIID Ebola virus challenge material utilized for these studies (Ebola virus/H.sapiens-tc/COD/1995/Kikwit-9510621) was produced following a total of 4 amplifications in Vero E6 cells. Prior to use, the stock was tested for purity (sterility testing, endotoxin testing, and mycoplasma testing) and identity (electron microscopy, inclusionary and exclusionary qRT-PCR analysis and deep sequencing). The stock was maintained at or below −70 °C until use.

For each exposure route cohort, three male and three female adult rhesus macaques (*Macaca mulatta*) of Chinese origin were obtained from the USAMRIID colony from licensed and approved vendors. For intramuscular exposure of virus, NHPs were challenged with a target dose of 1000 plaque-forming units (PFU) of EBOV in the right caudal thigh muscle using a 25 gauge needle. For the aerosol cohort, NHPs were exposed to a target dose of 1000 PFU of EBOV in a head-only chamber within a biosafety level 4 (BSL-4) laboratory containing a class III biological safety cabinet maintained under negative pressure. Prior to aerosol exposure, whole body head-out plethysmography (Buxco Research Systems, Wilmington, NC) was performed under anesthesia for computation of the minute volume and post-exposure calculations of the individual exposure doses. Animals were then individually exposed to virus in a 16 L head-only exposure chamber for a time-calculated exposure using aerosols generated by a three-jet Collision Nebulizer (BGI, Inc., Butler, NJ) controlled by an automated bioaerosol exposure (ABESII) system. Individual exposures were determined from samples collected in all-glass impingers.

#### NHP blood collection, processing, and storage

NHPs were monitored at least twice daily for clinical signs of illness. Animals were anesthetized with ketamine or telazol prior to physical examination and phlebotomy. For IM-challenged animals, blood was drawn prior to virus exposure at day −6 and day 0. Additional samples were obtained at day 3, 6, 10, 14, and at study endpoint/euthanasia. For the aerosol-challenged cohort, blood samples were collected prior to virus exposure at day −7 and day 0. Additional samples were collected at day 3, 6, and at euthanasia (day 7 and 8). Whole blood was collected in K_2_ EDTA tubes. EDTA plasma was prepared by centrifugation for 10 minutes at 3000 × *g* and plasma samples were mixed with 3 volumes of TRIzol LS (Life Technologies, Grand Island, NY) and stored at −80 °C. Serum was collected separately from whole blood in serum clot activator tubes (Vacuette, Grenier Bio-One North America, Inc., Monroe, NC) for chemistry analysis. Whole blood was collected into 3.2% sodium citrate tubes (Vacuette) for coagulation analysis.

### Hematology analysis, serum chemistry, and coagulation assays

Whole blood collected in K_2_ EDTA tubes was used for determination of quantitative blood cell counts with differential using the HemaVet 950FS Hematology Analyzer (Drew Scientific Inc., Miami, FL, USA) in accordance with manufacturer’s protocols. Serum chemistry values were obtained from ~100 μL samples run on a Piccolo General Chemistry 13 reagent disc using a Piccolo xpress Chemistry Analyzer (Abaxis, Union City, CA, USA). The IDEXX Coag Dx Analyzer (IDEXX Laboratories, Inc., Westbrook, ME) was used to evaluate prothrombin time (PT) and activated partial thromboplastin time (aPTT) using citrated whole blood.

### Human clinical serum/plasma samples

Fifteen human clinical serum or plasma samples (Supplemetary Table 1) were obtained from Kenema Government Hospital between June and August 2014 during the EBOV/Makona outbreak in the Republic of Sierra Leone, West Africa. The patient set consists of seven males and seven females, with ages ranging between 17 and 58 years (median age 39 years). Two samples from the same subject, taken 4 days apart, were included in this set. Patients were classified as EBOV-positive using fielded diagnostic assays^65^. Date of symptom onset was recorded based on patient self-reporting. Of the fourteen individuals, 10 survived the disease and were later discharged after negative viral RT-PCR and antigen tests. Three individuals did not survive, and one could not be located for follow-up.

### Total RNA extraction from TRIzol-inactivated plasma

Total RNA extractions from both NHP and human samples were performed using a previously optimized spin column-based method^66^. Briefly, archived aliquots of 100 μL plasma and 300 μL TRIzol LS, and 50 μL human serum/plasma samples mixed with 150 μL TRIzol LS were processed, adjusted to final volume of 1 mL. Glycogen (5 μg, from Life Technologies) was added, along with synthetic short RNAs (1 μL) from the RNA spike-in kit (Exiqon, Inc., Woburn, MA). Chloroform (150 μL) was added, and each tube was vortexed vigorously for 30 seconds then incubated at room temperature for 3 minutes. Phase separation was achieved by centrifuging the sample for 12,000 × *g* for 15 minutes at 4 °C, and 400 μL of the aqueous phase purified using the miRNeasy Mini Kit (QIAGEN, Inc., Valencia, CA).

### Viral load determination using RT-PCR

Viral loads from cell-free blood samples were determined with RT-PCR using an internally validated TaqMan probe assay targeting an 80-bp sequence in the glycoprotein^11^ shown to amplify both EBOV/Kikwit and EBOV/Makona^67^. The forward primer sequence is 5′-TTTTCAATCCTCAACCGTAAGGC-3′, the reverse primer sequence is 5′-CAGTCCGGTCCCAGAATGTG-3′, and the probe sequence is 6FAM-CATGTGCCGCCCCATCGC TGC–TAMRA-3′. Reactions were composed of the given primer and probe, as well as reagents from Invitrogen SuperScript™ One-Step RT-PCR Kit plus bovine serum albumin and 3 mM MgSO_4_. The following cycling conditions were used: 50 °C for 15 minutes (1 cycle); 95 °C for 5 minutes (1 cycle); 95 °C for 1 second and 60 °C for 20 seconds (45 cycles); and 40 °C for 30 seconds (1 cycle). A single fluorescence read was taken at the end of each 60 °C step. A standard curve was created from serial dilutions of the EBOV/Kikwit challenge stock (provided as PFU/mL based on plaque assay) extracted identically to the plasma samples, and quantification cycle (Cq) values were calculated using the second derivative method implemented in the integrated Roche LightCycler 480 software version 1.5.1.

### cDNA synthesis and miRNA profiling

Extracted RNA was reverse-transcribed with the cDNA Synthesis Kit II using 8 μL RNA for each 40 μL sample. cDNA samples were pooled and mixed with ExiLENT SYBR Green master mix, and 10 μL of the mixture was used in each well of the microRNA Ready-to-Use PCR Human panel I+II V3.R (all reagents from Exiqon, Inc., Woburn, MA). Amplifications were performed in triplicate on a LightCycler 480 II (Roche, San Francisco, CA) using manufacturer-recommended cycling conditions: 95 °C for 10 min, 45 cycles of 95 °C for 10 s, 60 °C for 1 min with a ramping rate of 1.6 °C/s, and a final melt-curve analysis.

### Data processing and analyses

Cq values were obtained using the second derivative method within the integrated Roche LightCycler 480 software version 1.5.1. GenEx 6 Enterprise software (MultiD Analyses AB, Göteborg, Sweden) was used to pre-process PCR panel data. Interplate calibrations were performed using the entire dataset for each cohort, followed by removal of samples with Cq > 38 and miRNAs that were not detected in 4 out of 6 NHPs. In human samples, all miRNAs with Cq > 38 were excluded from analyses. miRNA levels were normalized to the mean signal of all detected sequences with Cq < 36 per plate (global normalization^68^), and these ΔCq values were log_2_ transformed and used in subsequent analyses and data visualization.

### Determination of significant circulating miRNAs and pathway analysis

Significantly altered miRNAs over the infection timecourse were determined using the maSigPro package^33^ run in R version 3.1.0^69^. For both NHP groups, significant miRNAs were selected using a false discovery rate of 0.05 and a model fit value of R^2^ > 0.6. Data from the IM challenge was modeled with a quartic function (degree = 4) while a cubic function (degree = 3) was used for the aerosol data. Visual inspection of data profiles was used to determine polynomial regression degrees.

DNA Intelligent Analysis (DIANA)-mirPath v3.0^41^ was used to determine mRNA targets of significant miRNAs as well as to calculate overrepresented biological pathways by computing p-values that describe Kyoto Encyclopedia of Genes and Genomes (KEGG) pathway enrichment of predicted mRNA targets.

### miRNA-based classifier of EBOV infection

Variables for the prediction of EBOV infection were determined using machine learning, implemented in Partek Genomics Suite version 6.6 (St. Louis, MO). K-nearest neighbor, discriminant analysis, nearest centroid, support vector machine, and random forest algorithms were tested for classifier selection. For NHP groups, samples were designated as infected from day 3 (presymptomatic stage) through terminal timepoints. For human data, samples were categorized based on viral load; patients with viral counts >1000 PFU/mL were considered acutely infected for classifier selection and validation. Each of the three datasets was used separately for classifier training. The top 2–3 models of each type of classifier which categorized the training set with the highest accuracy were validated separately with the remaining two datasets. The most accurate classifier from these trials was then tested with samples from the remaining two datasets combined and randomized. The top scoring models and associated classifier metrics are given in Supplementary Table S4.

## Additional Information

**How to cite this article**: Duy, J. *et al.* Circulating microRNA profiles of Ebola virus infection. *Sci. Rep.* **6**, 24496; doi: 10.1038/srep24496 (2016).

## Supplementary Material

Supplementary Information

Supplementary Table S2

Supplementary Table S3

Supplementary Table S4
