# Supplementary material for: Circulating microRNA profiles of Ebola virus infection
Source: Sci Rep. 2016 Apr 21;6:24496. doi: 10.1038/srep24496 (PMC4838880; doi:10.1038/srep24496)

Circulating microRNA profiles of Ebola virus infection

Janice Duy[a], Jeffrey W. Koehler[a], Anna N. Honko[b#], Randal J. Schoepp[a], Nadia Wauquier[c], Jean-Paul Gonzalez[d], M. Louise Pitt[b], Eric M. Mucker[b], Joshua C. Johnson[b#], Aileen O'Hearn[a], James Bangura[c], Moinya Coomber[c], Timothy D. Minogue[a*]

Diagnostic Systems Division, U.S. Army Medical Research Institute of Infectious Diseases, Fort Detrick, Frederick, MD, USA[a]; Virology Division, U.S. Army Medical Institute of Infectious Diseases, Fort Detrick, Frederick, MD, USA[b]; Metabiota, Kenema, Sierra Leone[c]; Metabiota, Washington, DC, USA[d]

[*]Address correspondence to Timothy D. Minogue, timothy.d.minogue.civ@mail.mil

[#]Current address: Integrated Research Facility, National Institute of Allergy and Infectious Diseases, National Institutes of Health, Fort Detrick, Frederick, MD, USA

Supplementary Table S1. Patient characteristics for human serum/plasma samples used in the study.

Supplementary Figure S1. Kaplan-Meier hourly survival curve for Ebola virus-infected NHPs.

Supplementary Figure S2. Serum chemistry values during Ebola virus challenge of rhesus macaques. Normal ranges (shown as grey shaded areas as applicable) are the mean ± 2SD of repeated baseline values of rhesus macaques from the USAMRIID colony (n=119; 57 male). Samples were collected and processed similarly to this study and performed on the same chemistry and hematology systems. These capture 95% of the normal heathy range.

Supplementary Table S2. Relative expression values of significant miRNAs in IM-challenged NHPs. Significant miRNAs were selected using two-step regression. Values were globally normalized to the mean of each PCR plate and $\log_2$ transformed.

Supplementary Table S3. Relative expression values of significant miRNAs in aerosol-challenged NHPs. Significant miRNAs were selected using two-step regression. Values were globally normalized to the mean of each PCR plate and $\log_2$ transformed.

Supplementary Table S4. Ebola infection host miRNA classifier training and testing results.

Supplementary Table S1. Patient characteristics for human serum/plasma samples used in the study.

Patient designator	Day post symptom onset	Outcome	Age	Sex	Sample type	District
G1	2	Dead	54	F	Plasma	Kenema
G2	2	Missing	17	M	Serum	Moyamba
G3	3	Dead	30	F	Serum	Kenema
G4	4	Alive	50	M	Serum	Kenema
G5	4	Alive	50	F	Serum	Kenema
G6	8	Dead	50	M	Plasma	Kenema
G7	10	Alive	40	F	Serum	Kailahun
G8	13	Alive	45	M	Serum	Kailahun
G9	13	Alive	38	F	Serum	Kenema
G10	13	Alive	51	M	Serum	Kenema
G11	13	Alive	18	F	Plasma	Kenema
G12	15	Alive	27	M	Serum	Port Loko
G8	17	Alive	45	M	Serum	Kailahun
G13	18	Alive	20	F	Serum	Kenema
G14	27	Alive	17	M	Serum	Kailahun

Supplementary Figure S1. Kaplan-Meier hourly survival curve for EBOV-infected NHPs.

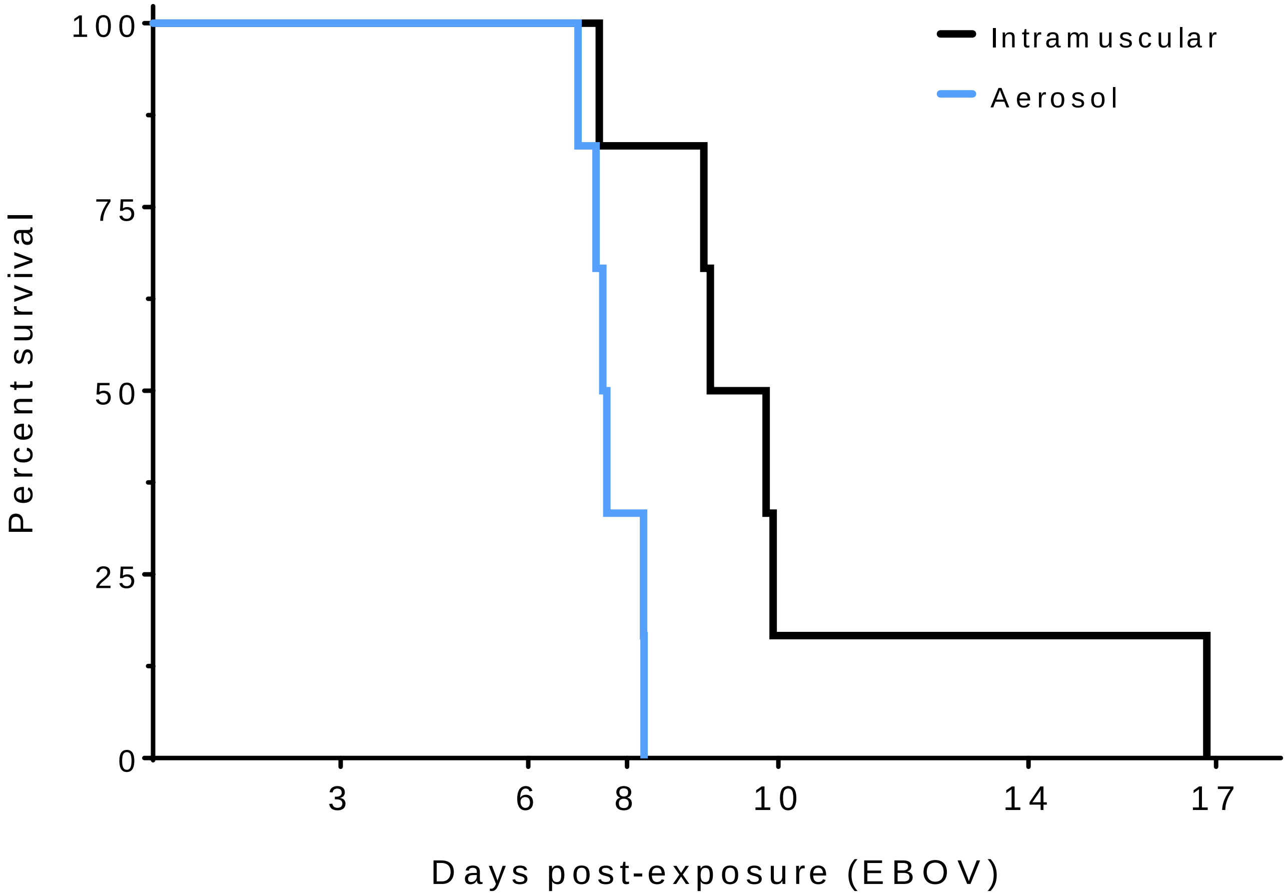

Supplementary Figure S2. Serum chemistry values during EBOV challenge of rhesus macaques. Normal ranges (shown as grey shaded areas as applicable) are the mean ± 2SD of repeated baseline values of rhesus macaques from the USAMRIID colony (n=119; 57 male). Samples were collected and processed similarly to this study and performed on the same chemistry and hematology systems. These capture 95% of the normal heathy range.

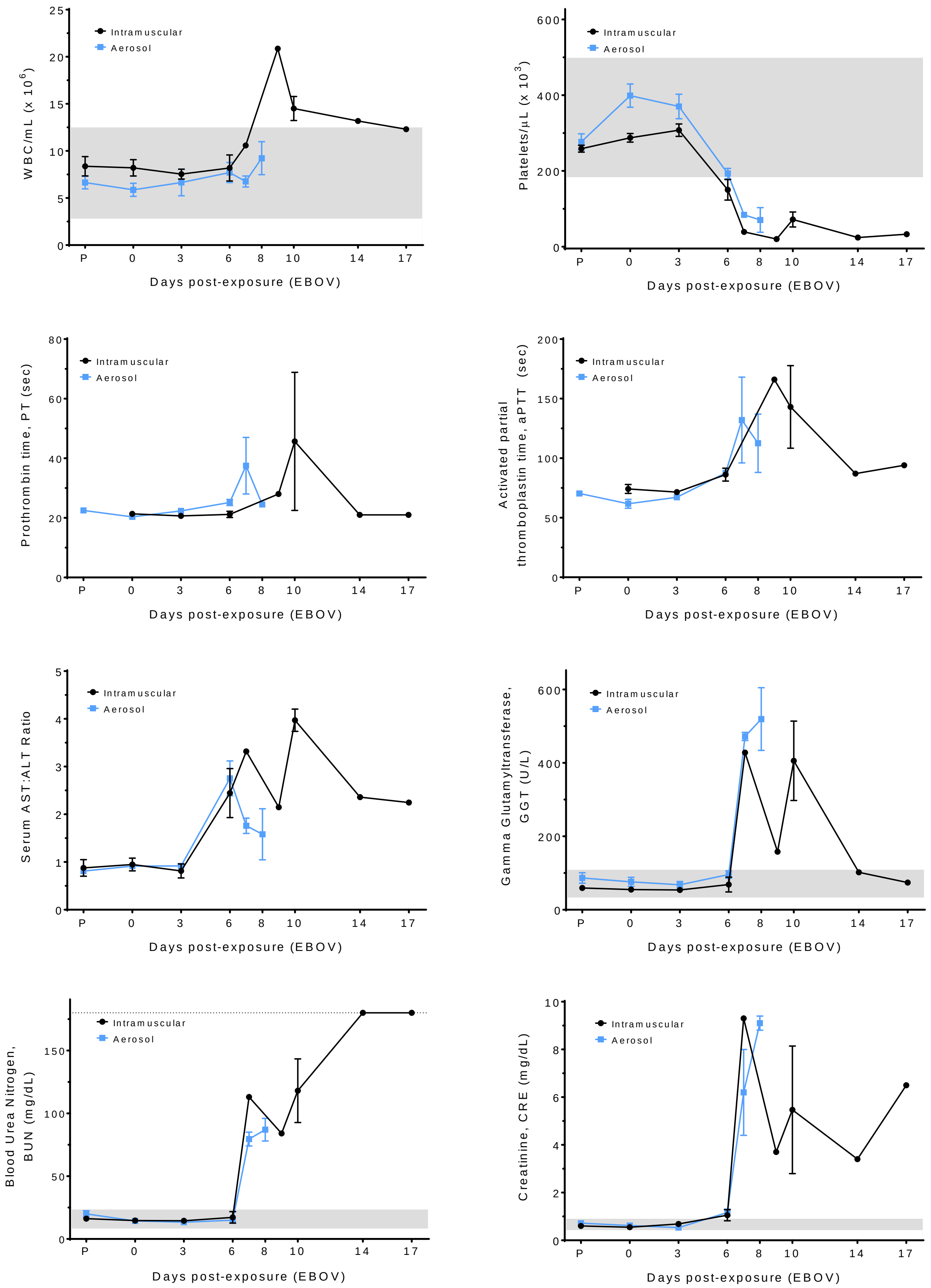

Supplement: Supplementary Information [file srep24496-s1.pdf]
